# SOX9-transactived long non-coding RNA NEAT1 promotes the self-renewal of liver cancer stem cells through PKA/Hippo signaling

**DOI:** 10.1038/s41392-021-00466-x

**Published:** 2021-02-26

**Authors:** Zhuo Cheng, Xijun Liang, Cheng Zhang, Ruoyu Wang, Tingting Wei, Beifang Ning, Elzbieta Poreba, Liang Li, Hongyang Wang, Jin Ding

**Affiliations:** 1 International Cooperation Laboratory on Signal Transduction, Eastern Hepatobiliary Surgery Hospital/Institute, The Second Military Medical University, Shanghai, China; 2 Department of Hepatic Surgery, Eastern Hepatobiliary Surgery Hospital, The Second Military Medical University, Shanghai, China; 3 Department of Laboratory Medicine, Changzheng Hospital, The Second Military Medical University, Shanghai, China; 4 Department of Gastroenterology, Changzheng Hospital, The Second Military Medical University, Shanghai, China; 5 Department of Molecular Virology, Institute of Experimental Biology, Faculty of Biology, Adam Mickiewicz University, Poznań, Poland; 6 National Center for Liver Cancer, Shanghai, China; 7 Clinical Cancer Institute, Center for Translational Medicine, Second Military Medical University, Shanghai, 200433 China

**Keywords:** Cancer stem cells, Gastrointestinal cancer

**Dear Editor,**

Hepatocellular carcinoma (HCC), the most common pathological type of primary liver cancer, ranks as the third deadliest cancer. Despite the progress of surgical resection in recent years, the 5-year survival of HCC patients is still unsatisfactory due to the frequent relapse and chemoresistance. Accumulating evidence has demonstrated that liver cancer stem cells (CSCs) are critical for HCC chemoresistance and recurrence. Nevertheless, the molecular mechanisms of liver CSC regulation remain unclear, which hampers the development of the therapeutic strategy that targets liver CSCs.

Long non-coding RNAs (LncRNAs) are defined as RNA transcripts >200 nucleotides (nt) in length without protein-coding potential, which have been reported to be involved in organ development, stemness maintenance, and cell differentiation in physiological processes, but the role of lncRNAs in liver CSC expansion remains unclear. Since self-renewal and chemoresistance are distinct characteristics of CSCs, we enriched liver CSCs by inducing hepatoma spheroid formation in the presence of cisplatin, and an lncRNA microarray analysis identified 87 lncRNAs upregulated in liver CSCs (Supplementary Fig. s1a and Table s1), among which the nuclear paraspeckle assembly transcript 1 (NEAT1) is the most prominent lncRNA in cancer research. We then compared the expression of NEAT1 in HCC spheres and attached HCC cells by fluorescence in situ hybridization and observed the evident upregulation of NEAT1 in HCC spheres (Fig. 1a). Epithelial cell adhesion molecule (EpCAM), cluster of differentiation 24 (CD24), and oval cell 6 (OV6) have been reported to serve as surface biomarker of liver CSCs.^1^ Herein upregulated NEAT1 expression was detected in EpCAM-, CD24-, or OV6-positive HCC cells, which further suggested that NEAT1 is highly expressed in liver CSCs (Figs. 1b and s1b). In addition, a positive correlation between NEAT1 and liver CSC markers was also observed in patient HCC tissues (Supplementary Fig. s1c). High NEAT1 expression was observed in the majority of patient HCCs (56/81) compared to the cancer adjacent tissues (cohort 2), while no population distribution difference in gender or age was found between the NEAT1 high and NEAT1 low patient groups (data not shown). Moreover, the patients with high NEAT1 expression had a higher recurrence rate than did patients with low NEAT1 expression (Supplementary Fig. s1d).

**Fig. 1:**
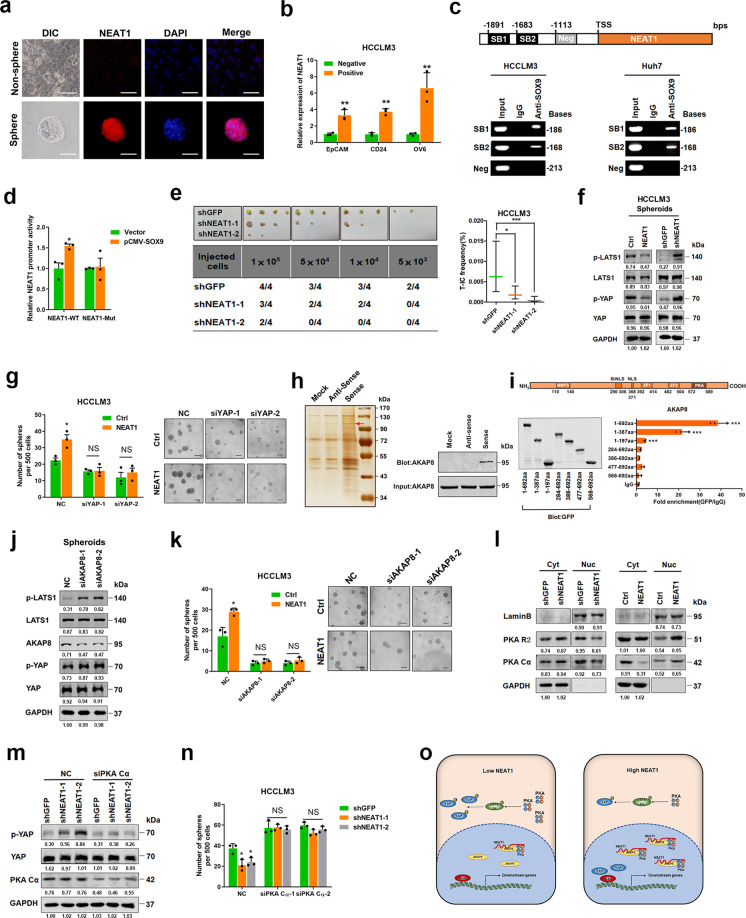
SOX9-transactived long non-coding RNA NEAT1 promotes the self-renewal of liver cancer stem cells through PKA/Hippo signaling. **a** The expression of NEAT1 in adherent and spheroid Huh7 cells was confirmed by FISH assays. Scale bar = 50 μm. **b** qRT-PCR analysis of NEAT1 in sorted EpCAM-, CD24-, or OV6-positive HCCLM3 cells relative to negative cells. **c** ChIP assay of the enrichment of SOX9 on NEAT1 promoter relative to IgG in HCC cells. SB indicates the SOX9-binding site and Neg indicates the region of negative control. **d** HCCLM3 cells were transfected with NEAT1-WT luciferase reporter plasmid or NEAT1-Mut luciferase reporter plasmid, together with pCMV-SOX9 or vector plasmid, and then subjected to luciferase reporter assay. Data were normalized against Renilla luciferase activity. **e** In vivo limiting dilution assay of HCCLM3 NEAT1-knockdown and control sphere-derived cells. Data are shown as the mean ± 95% CI, *n* = 4 for each group. **f** Phosphorylation of LATS1 and YAP in HCCLM3 NEAT1-overexpressing spheroids (left) and NEAT1-knockdown spheroids (right) was determined by western blot. **g** Sphere-formation assay of NEAT1-overexpressing cells transfected with two independent siRNAs targeting YAP. Scale bar = 100 μm. **h** RNA pulldown assays were performed with lysates of HCC spheres using full-length NEAT1 and antisense RNA probes, followed by mass spectrometry (left). Red arrows indicate the target band. Western blot analysis of AKAP8 in RNA pulldown precipitates retrieved by biotin-labeled NEAT1 or antisense RNA from the lysates of HCC spheres (right). **i** Deletion mapping for the domains of AKAP8 (upper). RIP analysis for NEAT1 enrichment in cells transiently transfected with plasmids containing the indicated EGFP-tagged full-length or truncated constructs (down). **j** Phosphorylation of LATS1 and YAP was determined by western blot after knockdown of AKAP8 in HCCLM3 spheroids. **k** The sphere-formation assay of HCCLM3 NEAT1-overexpressing cells transfected with two independent siRNAs targeting AKAP8. Scale bar = 100 μm. **l** Western blot analysis of PKA R2 and PKA Cα in subcellular fractions of HCCLM3 NEAT1-knockdown spheroids (left) and NEAT1-overexpressing spheroids (right). GAPDH and Lamin B acted as cytoplasm and nucleus markers, respectively. **m** Phosphorylation of YAP was determined by western blot after knockdown of PKA Cα in HCCLM3 NEAT1-knockdown cells. **n** The sphere-formation assay of HCCLM3 NEAT1-knockdown cells transfected with two independent siRNAs targeting PKA Cα. Scale bar = 100 μm. **o** The schematic model of the mechanism underlying the role of NEAT1 in LCSC expansion. *P < 0.05, **P < 0.01, and ***P < 0.001

Although NEAT1 has been demonstrated to be highly expressed in various cancer types, the mechanism underlying its upregulation is not clear. Bioinformatics analysis revealed two conserved binding sites of stemness-associated transcription factor SOX9 (SRY-box transcription factor) in the promoter region of NEAT1 (Fig. 1c). Chromatin immunoprecipitation assay demonstrated that SOX9 was enriched on both two predicted SOX9-binding sites in NEAT1 promoter (Fig. 1c). We then constructed luciferase reporter plasmids of NEAT1 promoter containing wild-type SOX9-binding sites (NEAT1-WT) or mutant SOX9-binding sites (NEAT1-Mut). As expected, the NEAT1-Mut luciferase reporter could not be activated by SOX9 overexpression (Fig. 1d). As shown in Fig. s2a, transfection of SOX9-expressing plasmid robustly increased the NEAT1 transcripts, which further confirm the transactivating role of SOX9 in NEAT1 expression. Consistently, a positive correlation between NEAT1 expression and SOX9 levels was observed in patient HCC tissues (Supplementary Fig. s2b). Considering NEAT1−/− mice was reported to express less Sox9 compared with WT control,^2^ there could be a feedback regulation between SOX9 and NEAT1, which is worthy of future investigation.

To explore the role of NEAT1 in liver CSCs, NEAT1-silenced HCCLM3 and Huh7 cell lines were established (Supplementary Fig. s3a). Flow cytometric analysis demonstrated that interference of NEAT1 dramatically reduced the proportion of liver CSCs in HCC cells (Supplementary Fig. s3b). Moreover, NEAT1-silenced HCC cells formed fewer and smaller spheroids than did the control cells (Supplementary Fig. s3c, d). An in vitro limiting dilution assay illustrated that NEAT1 knockdown dramatically decreased the CSC population in hepatoma cells (Fig. s3e). Moreover, hepatoma cells expressing shNEAT1 displayed an attenuated tumor-initiation capacity in NOD-SCID mice compared with control cells (Figs. 1e and Supplementary Fig. s3f), further indicating the role of NEAT1 in liver CSC self-renewal. To further confirm the role of NEAT1 in liver CSCs, NEAT1-expressing transfectants were established (Supplementary Fig. s4a) and the consistent results were achieved (Supplementary Fig. s4b–g). Since certain drugs targeting RNA have been approved for clinic application, we propose that NEAT1 might be a promising therapeutic target in HCC therapy.^3^

To explore the molecular mechanism underlying NEAT1-mediated liver CSC self-renewal, a luciferase reporter assay was performed. Overexpression of NEAT1 activated the Hippo signaling pathway but did not influence the signal transducer and activator of transcription 3, SMAD family member 3, or β-catenin signaling cascades (Supplementary Fig. s5a). Hippo pathway plays an inimitable role in liver development, regeneration, and carcinogenesis.^4^ Herein phosphorylation of large tumor-suppressor kinase (LATS) and yes-associated protein (YAP), two key nodes in the Hippo pathway, was decreased by NEAT1 overexpression but increased by NEAT1 interference in HCC spheroids (Figs. 1f and Supplementary Fig. s5b), while phosphorylation of macrophage stimulating 1/2 was not altered (data not shown). Consistently, YAP downstream target genes cysteine rich 61 and connective tissue growth factor were induced by NEAT1 overexpression but reduced by NEAT1 interference (Supplementary Fig. s5c). NEAT1 overexpression significantly promoted the nuclear translocation of YAP in HCC cells (Supplementary Fig. s5d), which was further confirmed by nucleus YAP analysis in NEAT1-overexpressing or NEAT1-silenced HCC cells (Supplementary Fig. s5e). As expected, xenografted tissue derived from NEAT1-overexpressing HCC cells exhibited enhanced nuclear accumulation of YAP (Supplementary Fig. s5f). Importantly, our data showed that the interference of YAP blocked NEAT1-enhanced HCC spheroid formation (Figs. 1g and Supplementary Fig. s5g), suggesting that NEAT1 promotes liver CSC self-renewal through Hippo signaling.

To dissect the molecular mechanism of NEAT1-activated Hippo signaling in liver CSCs, RNA pulldown assay was performed and A kinase anchor protein 8 (AKAP8) was identified to interact with NEAT1 (Fig. 1h). RNA immunoprecipitation assay showed that NEAT1 was enriched in AKAP8 precipitates, which further confirmed the interaction between NEAT1 and AKAP8 (Supplementary Fig. s6a). To identify the NEAT1-binding domain of AKAP8, EGFP-tagged full-length and truncated AKAP8 plasmids were used. RNA immunoprecipitation assay suggested that AKAP8 could bind to NEAT1 through its N-terminal domain (Fig. 1i). In addition, we observed the partial colocalization between AKAP8 and NEAT1 by immunocytofluorescent staining (Supplementary Fig. s6b). Interestingly, phosphorylation of LATS1 and YAP was notably increased upon silencing AKAP8 (Fig. 1j), which was similar to the response observed upon NEAT1 interference. Although AKAP8 expression was not affected by NEAT1 knockdown or overexpression (Supplementary Fig. s6c), silencing AKAP8 dramatically blocked NEAT1-enhanced HCC cell spheroid formation (Figs. 1k and Supplementary Fig. s6d), suggesting that NEAT1 modulates Hippo signaling and liver CSC self-renewal through its interaction with AKAP8.

Given that AKAP8 has been reported to be a nuclear-specific cAMP-dependent protein kinase (PKA) anchor and retains PKA in the nucleus through its C-terminal domain, we hypothesized that PKA could be involved in NEAT1-mediated Hippo signaling activation and liver CSC self-renewal. We thereby determined the PKA distribution in HCC cells upon NEAT1 knockdown or overexpression. Our data showed that the nuclear translocation of PKA subunits was reduced by NEAT1 knockdown and increased by NEAT1 overexpression in HCC spheres (Fig. 1l). It is known that PKA could activate LATS and thereby enhances the phosphorylation of YAP.^5^ Herein we found that interfering with PKA expression abrogated the NEAT1 knockdown-mediated enhancement of YAP phosphorylation (Fig. 1m). Reduction of HCC cell spheroid formation upon NEAT1 knockdown was blocked after PKA Cα silence (Figs. 1n and Supplementary Fig. s7a) and overexpression of PKA Cα blocked NEAT1-enhanced HCC cell spheroid formation (Supplementary Fig. s7b). Nuclear YAP accumulation was also observed in NEAT1 highly expressed HCC tissues (Supplementary Fig. s8a). As expected, the levels of YAP target genes were associated with the expression of NEAT1 in the patient HCCs (Supplementary Fig. s8b). Taken together, our data suggest that NEAT1 interacts with AKAP8 and regulates the PKA/YAP signaling cascade to control the self-renewal of liver CSCs (Fig. 1o). Clinical investigation revealed that HCC patients with low NEAT1 levels could benefit from transcatheter arterial chemo-embolization treatment, while patients with high NEAT1 expression did not (Fig. s8c), which implies the predictive value of NEAT1 in HCC personalized therapy.

In summary, we found that NEAT1 is transactivated by stemness-associated factor SOX9 in liver CSCs. NEAT1 directly interacts with AKAP8/PKA and activates Hippo signaling to maintain the self-renewal of liver CSCs. Our results provide not only a promising therapeutic target against liver CSCs but also a potential predictor of patient benefit from chemotherapy.

## Supplementary information

Supplemental material
