# Supplementary material for: SOX9-transactived long non-coding RNA NEAT1 promotes the self-renewal of liver cancer stem cells through PKA/Hippo signaling
Source: Signal Transduct Target Ther. 2021 Feb 26;6:87. doi: 10.1038/s41392-021-00466-x (PMC7907135; doi:10.1038/s41392-021-00466-x)
Supplement: Supplementary file 1 — Supplemental material [file 41392_2021_466_MOESM1_ESM.doc]

**Supplementary Materials for**

**SOX9-transactived Long Non-coding RNA NEAT1 Promotes the Self-renewal of Liver Cancer Stem Cells through PKA/Hippo Signaling**

Zhuo Cheng^1,#^, Xijun Liang^1,#^, Cheng Zhang^1^, Ruoyu Wang^2^, Tingting Wei^3^, Beifang Ning^4^, Elzbieta Poreba^5^, Liang Li^1^, Hongyang Wang^1,6,*^ and Jin Ding^1,6,*^

^1^International Cooperation Laboratory on Signal Transduction, Eastern Hepatobiliary Surgery Hospital/Institute, the Second Military Medical University, Shanghai, China.

^2^Department of Hepatic Surgery, Eastern Hepatobiliary Surgery Hospital, the Second Military Medical University, Shanghai, China.

^3^Department of Laboratory Medicine, Changzheng Hospital, the Second Military Medical University, Shanghai 200003, China.

^4^Department of Gastroenterology, Changzheng Hospital, the Second Military Medical University, Shanghai, China.

^5^Department of Molecular Virology, Institute of Experimental Biology, Faculty of Biology, Adam Mickiewicz University in Poznan, Poland.

^6^National Center for Liver Cancer, Shanghai, China.

^#^These authors contributed equally to this work.

^*^Corresponding authors.

**This file includes:**

Materials and Methods

Figures. s1 to s8

Tables. S1 to S9

**Materials and Methods**

**Cell lines and reagents**

The human HCC cell line HCCLM3 was obtained from the Cell Bank of the Chinese Academy of Sciences (Shanghai, China). The human HCC cell line Huh7 was obtained from the Japanese Collection of Research Bioresources (JCRB, Japan). HEK-293T cells were purchased from the American Type Culture Collection (ATCC, Rockville, USA). All cells were cultured in DMEM (Gibco, USA) supplemented with 10% fetal bovine serum (Gibco, USA) at 37°C in a humidified incubator with 5% CO_2_. DMEM/F12, B-27 and insulin were purchased from Invitrogen (USA). EGF and bFGF were purchased from Peprotech (USA). The cells were checked daily for fungal, yeast and bacterial contamination by microscope. Mycoplasma contamination was routinely checked using mycoplasma PCR.

**Microarray analysis**

Liver CSCs from three HCC cell lines (Huh7, HepG2 and CSQT-2) were enriched in spheroids cultured with 1 μg/ml cisplatin for two weeks. Cisplatin (Sigma, USA) was dissolved in normal saline at around 25℃ and diluted into culture medium. The cells were then collected, and total RNA was extracted. The expression profile of lncRNAs was analyzed using a Human LncRNA Array v2.0 (ArrayStar). Three replicates for the liver CSCs among the distinct cell lines were included. Eighty-seven upregulated lncRNAs in liver CSCs from the three HCC cell lines are shown in Supplementary Table 1.

**Patients and Specimens**

All samples were surgically resected from patients at Eastern Hepatobiliary Surgery Hospital (Shanghai, China) from 2009 to 2012. Inclusion criteria included the following: no history of previous anticancer therapy; no history of other malignancies; complete resection of macroscopic liver tumors; and histopathologically proven HCC. Exclusion criteria were as follows: cholangiocarcinoma; tumors of uncertain origin or probable metastatic liver tumor; mixed type of primary liver cancer as confirmed histopathologically; and perioperative mortality. This study involved three cohorts of HCC patients: cohort 1 (76 patients), cohort 2 (81 patients) and cohort 3 (80 patients). Detailed clinicopathological features are described in Supplementary Table 2, Supplementary Table 3 and Supplementary Table 4. The patients with higher NEAT1 expression in cancer tissues compared to that in the adjacent noncancerous tissues was defined as NEAT1 high; and those patients exhibited lower NEAT1 expression in HCC tissues compared to that in the adjacent noncancerous tissues was defined as NEAT1 low.

**Reverse transcription PCR and quantitative real-time PCR**

Total RNA from the clinical tissue specimens and cell lines was extracted using TRIzol reagent (Invitrogen, USA). The cDNA synthesis was performed using the Reverse Transcription System (Promega, USA). Quantitative real-time PCR was performed using a SYBR Green PCR Kit (Applied Biosystems, USA) and LightCycler 960 Real-time PCR system (Roche, USA). The expression of the indicated genes was normalized to endogenous reference controls 18S and β-actin using the 2^-ΔΔCt^ method. The primers used are listed in Supplementary Table 5.

**Western blot analysis**

Cells were lysed with SDS lysis buffer containing protease inhibitors (Roche, USA). The protein concentrations were determined by the BCA (bicinchoninic acid) Protein Assay Kit (Pierce, USA). Proteins in the cell lysates were analyzed by immunoblot with primary antibodies and IRDye 800 CW-conjugated secondary antibody (Rockland Immunochemicals, USA). The fluorescence intensity was recorded using an Odyssey fluorescence scanner system (Li-Cor Biosciences, USA). The primary antibodies used in this study are listed in Supplementary Table 6.

**In situ hybridization (ISH)**

ISH for NEAT1 was performed by using a NEAT1 ISH probe Kit (Boster, China). Briefly, the sections were deparaffinized in xylene and rehydrated through a graded series of alcohol. Then, the sections were treated with 3% H_2_O_2_ at room temperature for 10 min to inactivate endogenous peroxidases. The sections were deproteinated using proteinase K diluted in 3% citric acid buffer for 30 min at 37°C. The slides were prehybridized at 38°C for 2-4 h and then hybridized with hybridization buffer containing probes targeting NEAT1 at 38°C overnight. Slides were then stringently washed in 2×SSC, 0.5×SSC and 0.2×SSC for 15 min each time and then blocked with blocking buffer for 30 min at 37°C. Sections were incubated with biotinylated digoxin solution, SABC and biotinylated peroxidase for 30 min in each incubation. The slides were incubated with diaminobenzidine (DAB) (Dako, USA) and counterstained with hematoxylin. High-resolution images were captured with an Aperio Scan Scope AT Turbo (Aperio, USA) equipped with Aperio Image Scope software (Aperio, USA). The staining was assessed based on the staining intensity and the percentage of positively stained cells using Image-Pro Plus 6.0 software (Media Cybernetics, Inc., USA).

**Fluorescence in situ hybridization**

NEAT1 fluorescence-conjugated probes kit was designed and purchased from RiboBio Biotechnology (China). The samples were treated under nondenaturing conditions followed by the addition of fluorescence-conjugated probes. For double FISH, primary and secondary antibodies were added. The samples were counterstained with DAPI and observed using confocal microscopy.

**Flow cytometry**

HCC cells were collected and washed with PBS. Each group of HCC cells was incubated with the 1 μg of EpCAM-APC antibody or isotype control antibody respectively for 30 min at 4°C in the dark. Samples were analyzed using a FACS apparatus MoFlo XDP (Beckman Coulter, USA). The independent gates from distinct groups incubated with isotype control antibody respectively were used for the further analysis.

**Sphere formation**

Five hundred single cells were seeded into 96-well ultra-low attachment culture plates (Corning, USA) in serum-free DMEM/F12 supplemented with B27 (1:50), 20 ng/ml EGF, 10 ng/ml bFGF, and 4 mg/ml insulin. One week later, the spheres were photographed and counted.

**Limiting dilution assay in vitro**

Cells were seeded into 96-well ultra-low attachment culture plates at various numbers (2, 4, 6, 8, 16, 32 and 64) and incubated for 7 days. CSC proportions were analyzed using ELDA software (http://bioinf.wehi.edu.au/software/elda/index.html) provided by the Walter and Eliza Hall Institute.

**Limiting dilution assay in vivo**

For the in vivo limiting dilution assay, spheroids were dissociated into single cells, serially diluted to the desired doses (5×10^3^, 1×10^4^, 5×10^4^ and 1×10^5^), and then subcutaneously injected into NOD-SCID mice. After two months, the number of tumors was counted, and the frequency of CSCs was assessed using ELDA software described as above.

**Luciferase reporter assay**

The luciferase reporter plasmids NEAT1-WT and NEAT1-Mut were constructed from Obio Technology. The YAP luciferase reporter plasmid (8xGTIIC-luciferase, #34615) was purchased from Addgene. Cells were co-transfected with luciferase reporter plasmids and a Renilla control plasmid (500:1) using Lipofectamine 2000 (Invitrogen, USA). Each group was run in triplicate in 48-well plates. Luciferase activity was detected by Synergy 2 Multidetection Microplate Reader (BioTek Instruments, Inc., USA) at 48 h after transfection. Renilla luciferase activity was used to normalize against the firefly luciferase activity.

**Chromatin immunoprecipitation**

ChIP assay was conducted using SimpleChIP® Enzymatic Chromatin IP Kit (Cell Signaling Technology) following the manufacturer’s instructions. Briefly, HCC cells were crosslinked with 1% formaldehyde at 37°C for 10 minutes and collected into PBS with PMSF. The samples were then subjected to ultrasound in ice bath to shear genomic DNA. Chromatins were immunoprecipitated by anti-IgG, anti-SOX9 antibodies and Protein G Magnetic Beads respectively. The DNA was then purified with EDTA, Tris pH 6.5 and glycogen. Fold enrichment was achieved by qRT–PCR and normalized to input chromatin. The primers for ChIP–quantitative PCR were listed in Supplementary Table 7.

**RNA pulldown**

NEAT1 or antisense NEAT1 RNAs were transcribed and labeled with a RNAmax-T7 Biotin-labeled Transcription Kit (RiboBio Biotechnology, China). Next, 3 μg of biotinylated RNA was pretreated with RNA structure buffer to obtain an appropriate secondary structure. The pretreated biotinylated RNAs were incubated with 1 mg of protein extracts from Huh7 cells at 4 °C for 1 h. Washed streptavidin agarose beads (Invitrogen, USA) were added to each binding reaction, all of which were incubated on a rotator overnight at 4 °C. Precipitates were washed three times, boiled in 60 μl of SDS buffer, separated by gel electrophoresis and visualized by silver staining. Specific bands were excised and analyzed by mass spectrometry. The NEAT1 primers for in vitro transcription are provided in Supplementary Table 8.

**RNA immunoprecipitation**

RIP assays were performed using the Magna RIP RNA-binding protein immunoprecipitation kit (Millipore, USA). Briefly, cells growing in 10-cm dishes were lysed in 1 ml of lysis buffer containing protease inhibitors and RNase Inhibitor (Thermo Fisher Scientific, USA). The supernatants were incubated with Protein G Dynabeads (Thermo Fisher Scientific, USA), which were incubated with the indicated antibodies overnight at 4°C with gentle rotation. The beads were washed three times with wash buffer containing RNase inhibitor and then twice with PBS containing RNase inhibitor. RNA extraction and qRT-PCR were performed as described above. Total RNA (input) and IgG controls were assayed simultaneously. For the RIP assays of deletion mutants, 8 μg of plasmids with EGFP-tagged full-length and truncated AKAP8 were transiently transfected into Huh7 cells, and the cell lysates were immunoprecipitated with the indicated antibodies. Information on the antibodies is listed in Supplementary Table 6.

**Nuclear and Cytoplasmic Protein Extraction**

Cytoplasmic and nuclear fractions of the HCC cells were prepared and collected according to the instructions of the Nuclear and Cytoplasmic Protein Extraction Kit (Beyotime, China). GAPDH was used as the cytoplasmic endogenous control. Lamin B was used as the nuclear endogenous control.

**Immunohistochemistry**

Tissues were fixed in 4% paraformaldehyde and embedded in paraffin. Serial paraffin sections were subjected to H&E staining and immunohistochemistry. Briefly, sections were deparaffinized with xylene, rehydrated, and then blocked with 1% BSA at room temperature for 30 min. After incubation with primary antibodies (YAP, Abcam, 1:100) at 4°C overnight, the sections were incubated with HRP-conjugated secondary antibodies at 37°C for 30 minutes. Positive reactions were visualized with DAB (Dako, USA) followed by hematoxylin counterstaining.

**Immunofluorescence**

HCC cells were plated in 15 mm glass bottom cell culture dishes at 30% confluence and allowed to grow for 24 h. Then, the cells were fixed with 10% paraformaldehyde solution for 15 min at room temperature, permeabilized with 0.4% Triton X-100 in PBS for 5 min, and then blocked with 1% BSA in PBS for 1 h at 37℃. The blocked cells were incubated with primary antibodies (YAP, Abcam, 1:100; AKAP8, Abcam, 1:100) overnight at 4°C, followed by incubation with Alexa Fluor 488-conjugated anti-rabbit IgG antibody (1:100, Invitrogen, Carlsbad, CA) for 2 h. Nuclear staining of the cells was conducted using DAPI. Representative images were acquired using a fluorescence microscope (IX70, Olympus, Japan).

**Small interfering RNA (siRNA) transfection and injection**

The sequences of small interfering RNA (siRNA) oligonucleotides targeting YAP, AKAP8, and PKA Cα as well as of the negative control siRNA are provided in Supplementary Table 9; they were purchased from RiboBio (RiboBio Biotechnology, China). Transfections with siRNA (100 nM) were performed with Lipofectamine 2000.

**Statistics**

All statistical analyses in this study were performed with SPSS 21.0 software (SPSS Inc., USA). Data are presented as the mean±s.d. The significance of the difference in the mean values between two groups was analyzed by two-tailed Student’s t-test. Pearson’s correlation analysis was performed to determine the correlation between two variables. Kaplan–Meier survival analysis was utilized to compare HCC patient survival based on dichotomized NEAT1 expression by the log-rank test. A *p-*value < 0.05 was considered significant.

**Supplementary Figures**

**
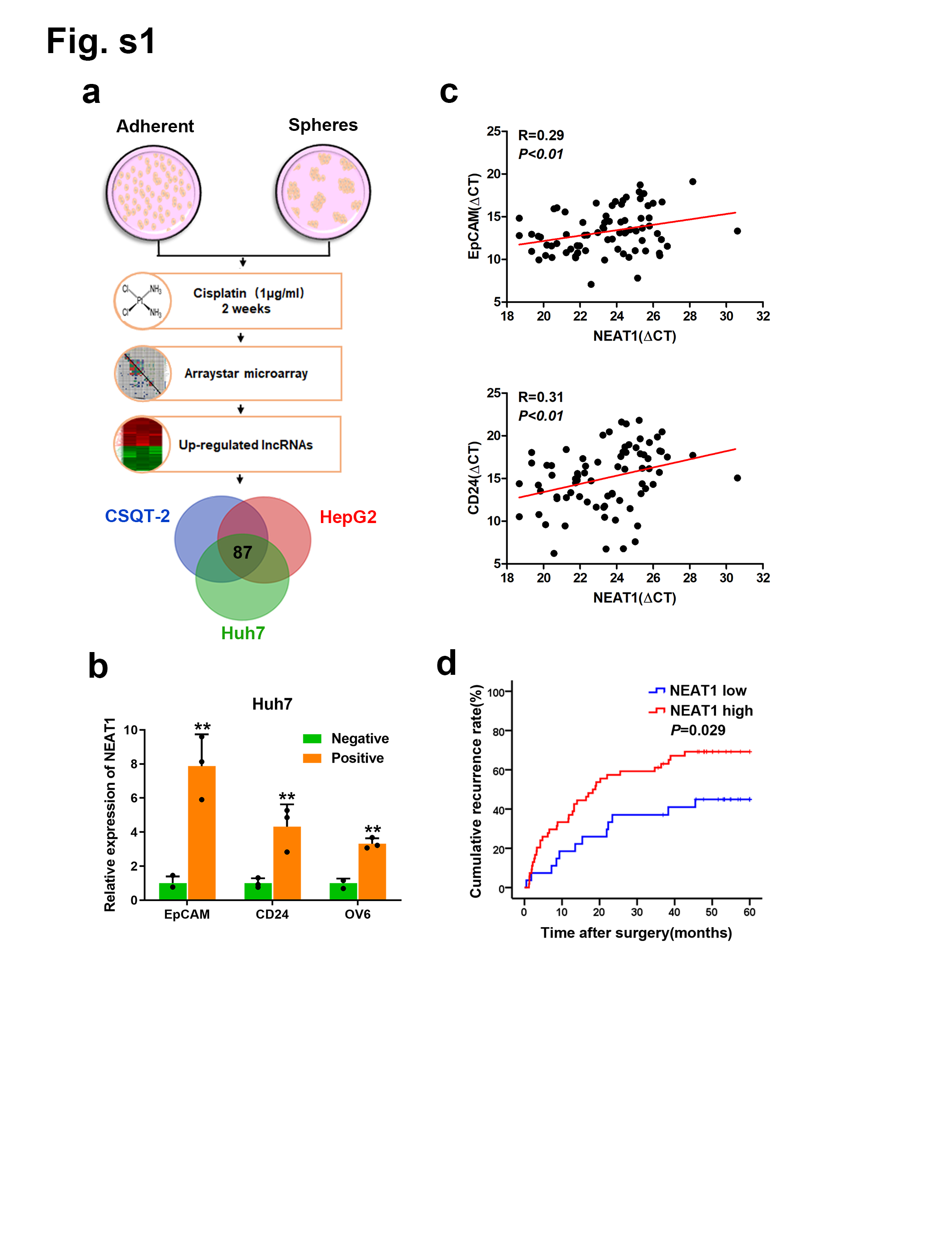
**

**Fig. s1. NEAT1 is highly expressed in liver CSCs.**

**a.** The flow chart for screening candidate lncRNAs in liver CSCs. **b.** qRT-PCR analysis of NEAT1 in MACS-sorted EpCAM, CD24 or OV6 positive Huh7 cells relative to negative cells. **c.** The correlation between the transcription level of NEAT1 and that of either EpCAM (upper) or CD24 down) in HCC tissues (cohort 1, n=76) was determined by qRT-PCR analysis. Data were normalized to 18s RNA, reported as ΔCt and analyzed by Pearson’s correlation analysis. **d.** Kaplan–Meier analysis the of recurrence rate of HCC patients with high or low NEAT1 expression in cohort 2 (n=81). “**” indicates P<0.01.

**
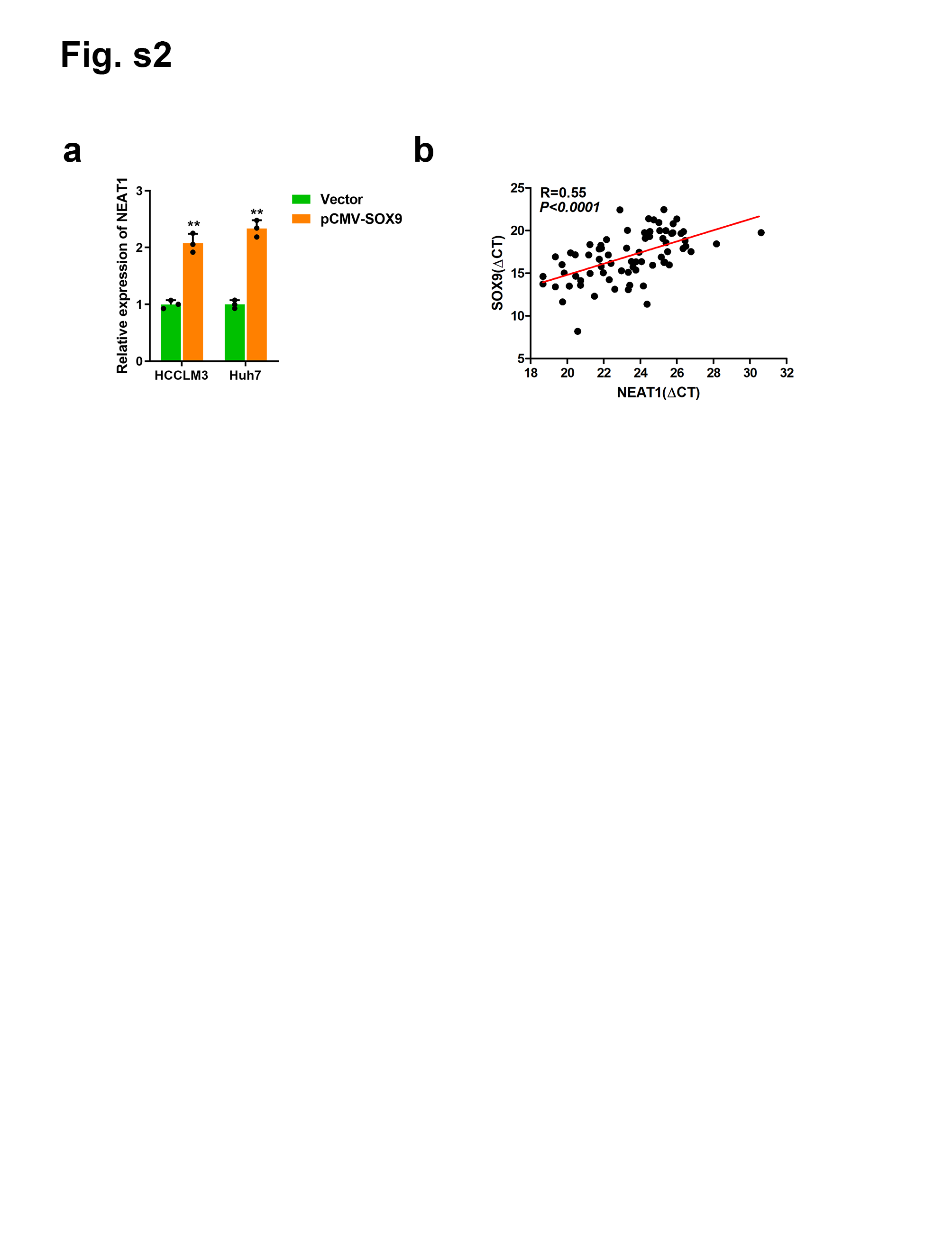
**

**Fig. s2. NEAT1 expression is transactivated by SOX9**

**a.** qRT–PCR analysis of NEAT1 in HCC cells transfected with pCMV-SOX9 plasmid or vector control after 48h. **b.** The correlation between NEAT1 and SOX9 in patient HCC tissues (cohort 1, n=76) was determined by qRT-PCR analysis. Data were normalized to 18s RNA, reported as ΔCt and analyzed by Pearson’s correlation analysis. “**” indicates P<0.01.

**
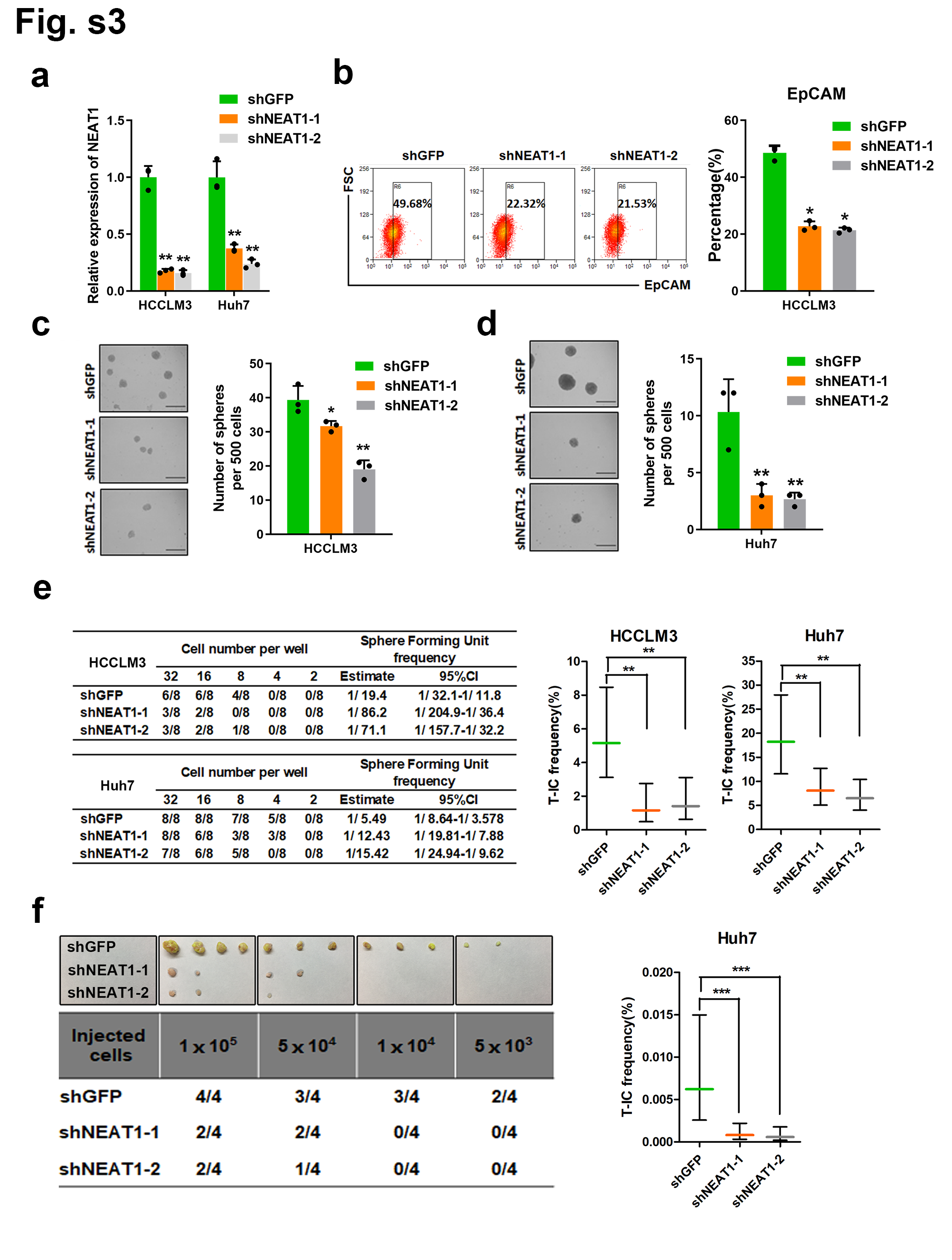
**

**Fig. s3. NEAT1 is required for liver CSC expansion.**

**a.** The expression of NEAT1 in NEAT1-knockdown and control HCC cells was measured by qRT-PCR. **b.** The proportion of EpCAM positive cells in HCCLM3 NEAT1-knockdown cells and control cells was detected by flow cytometric analysis. **c-d.** Sphere formation assay of NEAT1-knockdown cells and control cells. Scale bar=200 μm. **e.** The frequency of liver CSCs in NEAT1-knockdown and control HCC cells was compared by an in vitro limiting dilution assay. Data are shown as the mean±95% CI. **f.** In vivo limiting dilution assay of Huh7 NEAT1-knockdown and control sphere-derived cells. Data are shown as the mean±95% CI, n=4 for each group. “*” indicates *P* < 0.05, “**” indicates P<0.01, and “***” indicates P<0.001.


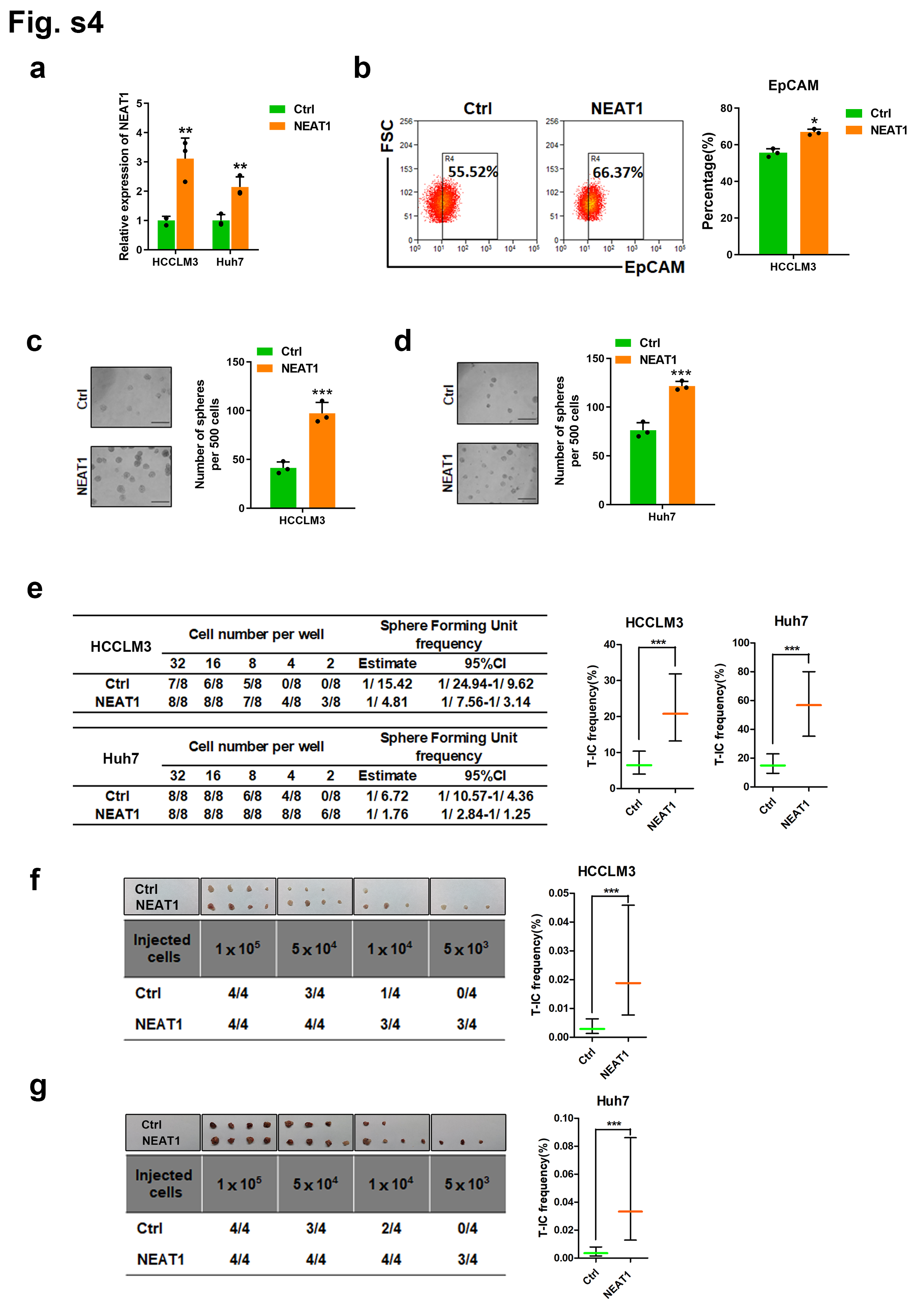


**Fig. s4. NEAT1 facilitates liver CSC expansion.**

**a.** The expression of NEAT1 in NEAT1-overexpressing and control HCC cells was measured by qRT-PCR. **b.** The proportion of EpCAM positive cells in HCCLM3 NEAT1-overexpressing cells and control cells were detected by flow cytometric analysis. **c-d.** Sphere formation assay of NEAT1-overexpressing cells and control cells. Scale bar=200 μm. **e.** The frequency of liver CSCs in NEAT1-overexpressing and control HCC cells was compared by an in vitro limiting dilution assay. Data are shown as the mean±95% CI. **f-g.** In vivo limiting dilution assay of NEAT1-overexpressing and control sphere-derived cells. Data are shown as the mean±95% CI, n=4 for each group. “*” indicates *P* < 0.05, “**” indicates P<0.01, and “***” indicates P<0.001.


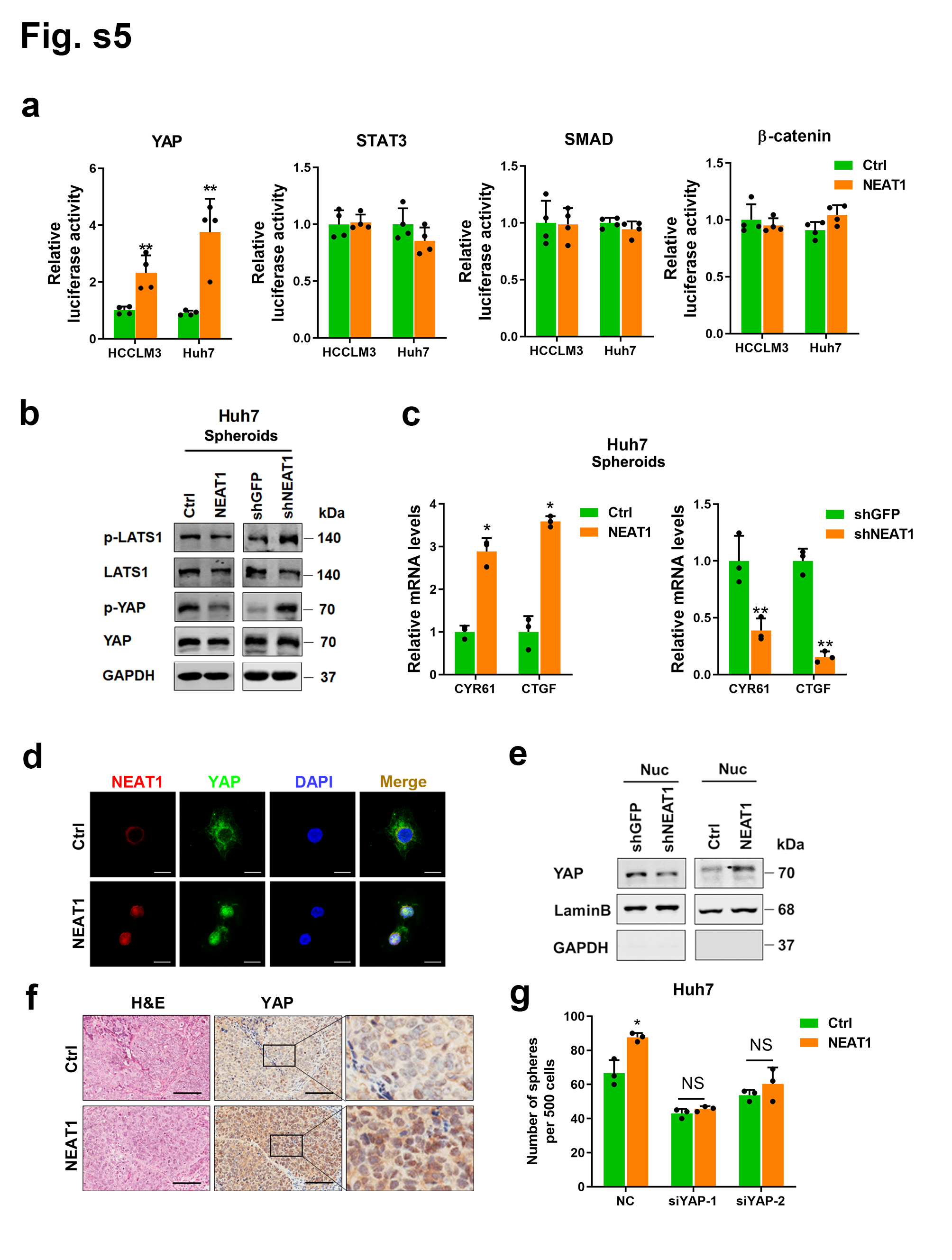


**Fig. s5. NEAT1 drives liver CSC self-renewal through Hippo signaling.**

**a.** HCCLM3 and Huh7 cells overexpressing NEAT1 and the corresponding control cells were transfected with YAP/TAZ, STAT3, SMAD or TOP/FOP(β-catenin) luciferase reporter plasmid and subjected to the luciferase reporter assay. Data were normalized against Renilla luciferase activity. **b.** Phosphorylation of LATS1 and YAP in Huh7 NEAT1-overexpressing spheroids (left) and NEAT1-knockdown spheroids (right) was determined by western blot. **c.** The expression of CYR61 and CTGF in Huh7 NEAT1-overexpressing spheroids (left) and NEAT1-knockdown spheroids (right) was analyzed by qRT-PCR. **d.** RNA FISH analysis of NEAT1 (red) and immunofluorescence detection of YAP (green) in HCCLM3 cells overexpressing NEAT1. Nuclear staining of the cells was conducted using DAPI (blue). Scale bar=7.5 μm. **e.** Western blot analysis of YAP in subcellular fractions of HCCLM3 NEAT1-knockdown spheroids (left) and NEAT1-overexpressing spheroids (right). GAPDH and Lamin B acted as cytoplasm and nucleus marker, respectively. **f.** Representative images of the immunohistochemical staining of YAP in NEAT1-overexpressing xenografted tumors. Scale bar=100 µm. **g.** Sphere formation assay of Huh7 NEAT1-overexpressing cells transfected with two independent siRNAs targeting YAP. “*” indicates P < 0.05, and “**” indicates P<0.01.


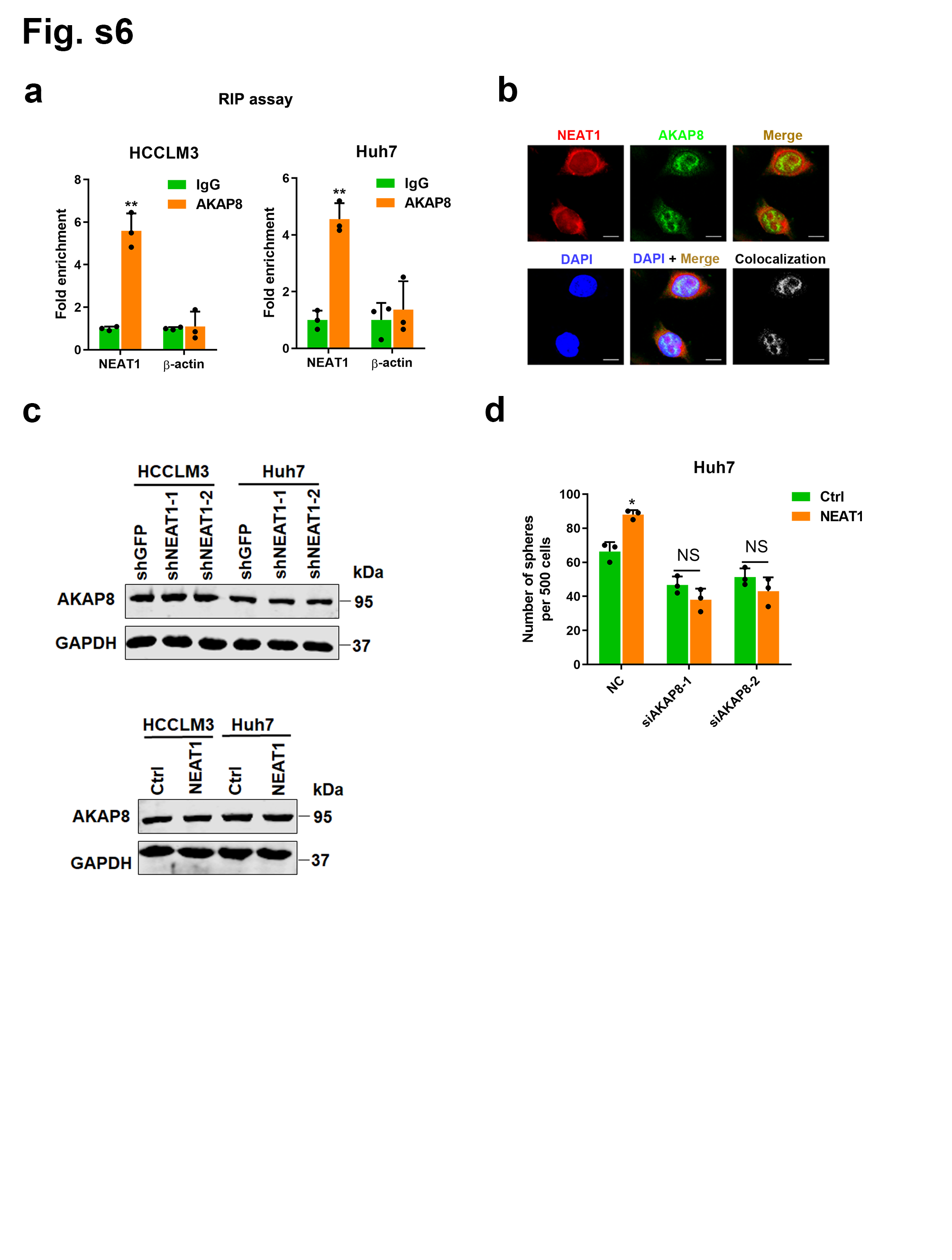


**Fig. s6. NEAT1 regulates Hippo signaling through its interaction with AKAP8.**

**a.** RIP assay of the enrichment of AKAP8 on NEAT1 relative to IgG in the lysates of HCC cells from spheres. β-actin served as a loading control. **b.** RNA FISH analysis of NEAT1 (red) and immunofluorescence detection of AKAP8 (green) in HCCLM3 cells. Nuclear staining of the cells was conducted using DAPI (blue). Scale bar=7.5 μm. **c.** Western blot analysis of AKAP8 expression in NEAT1-knockdown cells (upper) and NEAT1-overexpressing cells (down). **d.** The sphere formation assay of Huh7 NEAT1-overexpressing cells transfected with two independent siRNAs targeting AKAP8. “*” indicates P < 0.05, and “**” indicates P<0.01.

**
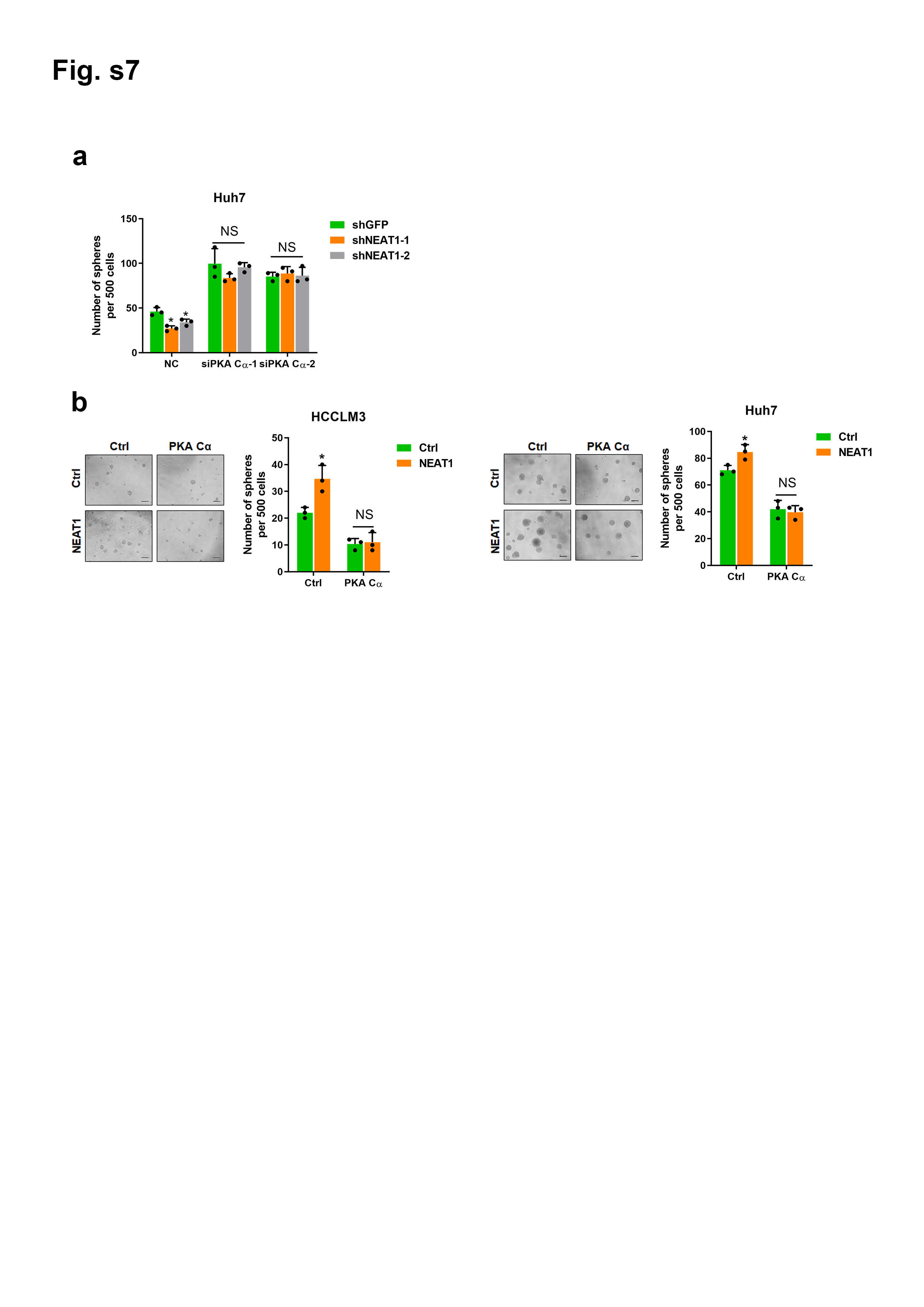
**

**Fig. s7. NEAT1 activates liver CSCs through the AKAP8/PKA axis.**

**a.** The sphere formation assay of Huh7 NEAT1-knockdown cells transfected with two independent siRNAs targeting PKA Cα. **b.** Spheres formation assay of NEAT1-overexpressing cells transfected with PKA Cα overexpression plasmid. Scale bar=100μm. “*” indicates *P* < 0.05.


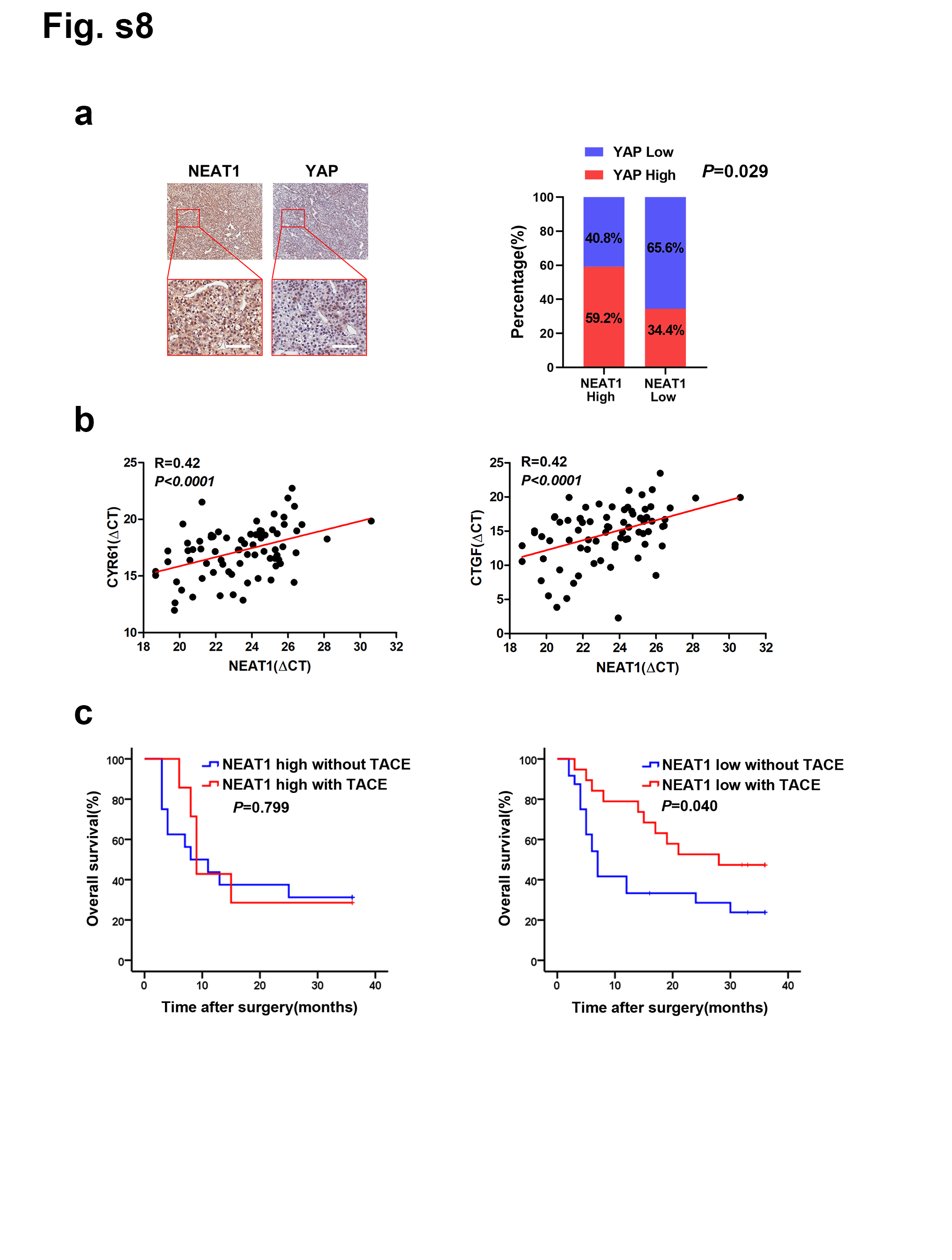


**Fig. s8. NEAT1 expression correlates with HCC malignancy and patient prognosis.**

**a.** Representative images of NEAT1 ISH and YAP immunohistochemistry (left) using consecutive sections in cohort 2 (n=81). Scale bar=100 μm. The correlation of NEAT1 and YAP expression was analyzed (right). **b.** The correlation between the transcription level of NEAT1 and CYR61 (left) or CTGF (right) in HCC tissues (cohort 1, n=76) was determined by qRT-PCR analysis. Data were normalized to 18s RNA, reported as ΔCt and analyzed by Pearson’s correlation analysis. **c.** Kaplan–Meier analysis of the overall survival of HCC patients with high (left) or low (right) NEAT1 expression, who had been treated with TACE or not after surgery in cohort 3 (n=80).

**Supplementary Tables**

**Supplementary Table 1.** Information of 87 up-regulated lncRNAs from microarray.

| **Genebank accession** | **Regulation** | **Source** | **Chr** | | **Fold change**  **(LCSCs/control > 2, p < 0.05)** | | |
| --- | --- | --- | --- | --- | --- | --- | --- |
|  |  |  |  |  | **Huh7** | **HepG2** | **CSQT-2** |
| ELF3-AS1 | Up | Ensembl | chr1 | 2.90 | | 4.15 | 31.06 |
| ANKRD36BP1 | Up | RefSeq_NR | chr1 | 6.37 | | 3.90 | 21.34 |
| AL138828.1 | Up | Ensembl | chr6 | 2.56 | | 11.43 | 21.25 |
| AK095453 | Up | misc_RNA | chr11 | 13.59 | | 2.61 | 17.21 |
| LINC02542 | Up | Ensembl | chr6 | 2.93 | | 2.64 | 10.61 |
| BX641027 | Up | misc_RNA | chr17 | 3.34 | | 3.49 | 10.02 |
| MR1 | Up | misc_RNA | chr1 | 6.71 | | 2.39 | 9.04 |
| AC017116.1 | Up | Ensembl | chr7 | 2.51 | | 2.50 | 8.96 |
| LOC105372833 | Up | Ensembl | chr21 | 2.66 | | 2.17 | 8.54 |
| AC011718.1 | Up | Ensembl | chr22 | 7.28 | | 2.72 | 7.73 |
| LINC01991 | Up | Ensembl | chr3 | 3.29 | | 5.34 | 7.63 |
| BC047481 | Up | UCSC_knowngene | chr2 | 4.45 | | 2.06 | 7.31 |
| AK289390 | Up | UCSC_knowngene | chr11 | 4.55 | | 2.32 | 7.13 |
| DHX40P1 | Up | RefSeq_NR | chr17 | 2.92 | | 2.39 | 6.36 |
| DKFZp451A185 | Up | UCSC_knowngene | chr18 | 2.81 | | 2.53 | 6.30 |
| AF130046.1 | Up | H-invDB | chr5 | 3.02 | | 2.01 | 5.58 |
| PPP1R3E | Up | RefSeq_NR | chr14 | 2.71 | | 2.55 | 5.20 |
| AC022483.1 | Up | Ensembl | chr4 | 2.00 | | 2.81 | 5.15 |
| AL024493.1 | Up | Ensembl | chrX | 2.76 | | 2.52 | 5.13 |
| LOC647859 | Up | RefSeq_NR | chr5 | 3.58 | | 2.14 | 5.13 |
| BX649153 | Up | misc_RNA | chr12 | 2.39 | | 2.18 | 4.95 |
| CR606969 | Up | misc_RNA | chr8 | 4.14 | | 3.09 | 4.89 |
| GGT3P | Up | Ensembl | chr22 | 7.58 | | 2.17 | 4.52 |
| PSMA3-AS1 | Up | RefSeq_NR | chr14 | 2.07 | | 2.99 | 4.32 |
| RP11-499O7.4 | Up | Ensembl | chr10 | 5.05 | | 2.78 | 4.28 |
| BC011766 | Up | NRED | chr3 | 2.81 | | 2.39 | 4.17 |
| uc.184 | Up | UCR | chr5 | 4.20 | | 2.57 | 4.06 |
| RWDD2A | Up | misc_RNA | chr6 | 3.16 | | 2.73 | 3.99 |
| CYB5D2 | Up | RefSeq_NR | chr17 | 2.91 | | 3.44 | 3.94 |
| AK056250 | Up | NRED | chr1 | 3.46 | | 2.82 | 3.92 |
| GPR157 | Up | misc_RNA | chr1 | 2.53 | | 2.55 | 3.90 |
| CXADRP1 | Up | Ensembl | chr21 | 3.79 | | 2.52 | 3.84 |
| HK2P1 | Up | Ensembl | chrX | 3.44 | | 2.68 | 3.65 |
| AL603832.1 | Up | Ensembl | chr1 | 2.51 | | 9.12 | 3.51 |
| BC044845 | Up | UCSC_knowngene | chr16 | 2.01 | | 2.34 | 3.48 |
| G43537 | Up | misc_RNA | chr2 | 2.65 | | 2.01 | 3.47 |
| BC018860 | Up | UCSC_knowngene | chr1 | 4.14 | | 2.31 | 3.45 |
| AK096317 | Up | NRED | chr16 | 2.12 | | 2.22 | 3.39 |
| AC079944.1 | Up | Ensembl | chr3 | 3.62 | | 2.21 | 3.37 |
| YWHAZP4 | Up | Ensembl | chr6 | 2.51 | | 3.26 | 3.35 |
| PSMD5-AS1 | Up | RefSeq_NR | chr9 | 2.37 | | 2.05 | 3.34 |
| AC010245.1 | Up | Ensembl | chr5 | 4.15 | | 2.23 | 3.32 |
| NEAT1 | Up | UCSC_knowngene | chr11 | 5.50 | | 2.34 | 3.28 |
| MIR22HG | Up | RefSeq_NR | chr17 | 2.76 | | 2.08 | 3.27 |
| LINC00652 | Up | RefSeq_NR | chr20 | 2.51 | | 5.86 | 3.19 |
| OR2A9P | Up | Ensembl | chr7 | 4.37 | | 2.05 | 3.17 |
| TSPAN8 | Up | lncRNA | chr12 | 4.69 | | 2.71 | 3.16 |
| CAMTA1-DT | Up | Ensembl | chr1 | 2.66 | | 3.81 | 3.04 |
| BC015064 | Up | misc_RNA | chr17 | 6.44 | | 3.35 | 3.00 |
| AK309762 | Up | UCSC_knowngene | chr1 | 2.07 | | 2.15 | 2.95 |
| AC013437.2 | Up | Ensembl | chr2 | 2.28 | | 3.06 | 2.90 |
| LOC100128288 | Up | RefSeq_NR | chr17 | 2.33 | | 2.70 | 2.87 |
| ITPRIPL2 | Up | RNAdb | chr16 | 2.79 | | 2.75 | 2.86 |
| U79272 | Up | NRED | chrX | 3.23 | | 2.76 | 2.74 |
| GCNT3 | Up | lncRNA | chr15 | 12.77 | | 2.11 | 2.73 |
| PSMA5 | Up | misc_RNA | chr1 | 2.85 | | 2.27 | 2.73 |
| AC010350.1 | Up | Ensembl | chr5 | 5.32 | | 2.01 | 2.73 |
| LMAN2L | Up | RefSeq_NR | chr2 | 2.58 | | 2.71 | 2.63 |
| NCRNA00120 | Up | RefSeq_NR | chr6 | 2.15 | | 2.01 | 2.62 |
| MFAP3 | Up | NRED | chr5 | 2.34 | | 2.05 | 2.56 |
| AL807761.3 | Up | Ensembl | chr9 | 2.12 | | 5.17 | 2.52 |
| LOC727916 | Up | misc_RNA | chr6 | 2.54 | | 2.14 | 2.48 |
| AL110159 | Up | RNAdb | chr6 | 2.45 | | 2.64 | 2.47 |
| AK124281 | Up | misc_RNA | chr6 | 2.61 | | 2.48 | 2.45 |
| FKBP9P1 | Up | Ensembl | chr7 | 2.37 | | 2.44 | 2.43 |
| AF052160 | Up | NRED | chr3 | 2.10 | | 5.63 | 2.38 |
| LOC152485 | Up | NRED | chr4 | 2.71 | | 2.43 | 2.36 |
| LOC150166 | Up | NRED | chr22 | 3.48 | | 2.12 | 2.33 |
| TMEM185A | Up | UCSC_knowngene | chrX | 3.87 | | 2.10 | 2.31 |
| IQSEC2 | Up | RefSeq_NR | chrX | 14.19 | | 2.62 | 2.30 |
| UBE2H | Up | UCSC_knowngene | chr7 | 3.50 | | 2.26 | 2.30 |
| PROS2P | Up | Ensembl | chr3 | 2.91 | | 2.45 | 2.30 |
| G36700 | Up | misc_RNA | chr16 | 2.91 | | 3.98 | 2.28 |
| AC093673.1 | Up | Ensembl | chr7 | 3.23 | | 2.00 | 2.25 |
| RRN3P1 | Up | RefSeq_NR | chr16 | 3.26 | | 2.62 | 2.24 |
| VNN3 | Up | Ensembl | chr6 | 16.99 | | 2.39 | 2.24 |
| SAT1 | Up | RefSeq_NR | chrX | 5.96 | | 2.14 | 2.22 |
| BC034791 | Up | misc_RNA | chr18 | 2.37 | | 2.21 | 2.15 |
| DL492557 | Up | UCSC_knowngene | chr10 | 2.12 | | 2.03 | 2.14 |
| AC012308.1 | Up | Ensembl | chr10 | 2.42 | | 2.27 | 2.12 |
| ATXN1 | Up | NRED | chr6 | 2.58 | | 2.05 | 2.12 |
| C1orf52 | Up | RefSeq_NR | chr1 | 2.57 | | 2.83 | 2.10 |
| HIF1A | Up | RNAdb | chr14 | 6.76 | | 2.09 | 2.10 |
| BC041923 | Up | UCSC_knowngene | chr19 | 2.06 | | 2.07 | 2.06 |
| MTCO1P5 | Up | Ensembl | chr3 | 3.37 | | 14.47 | 2.04 |
| MTND5 | Up | UCSC_knowngene | chrM | 2.49 | | 2.66 | 2.03 |
| LOC100288152 | Up | RNAdb | chr5 | 11.91 | | 2.46 | 2.02 |

**Supplementary Table 2. Clinicopathologic Features of 76 HCC Specimens (cohort 1).**

| **Characteristics** | |  | **Number of Patients** |
| --- | --- | --- | --- |
| Age(year) | ≤50 | | 27 |
|  | >50 | | 49 |
| Gender | Male | | 67 |
|  | Female | | 9 |
| HBsAg | Positive | | 65 |
|  | Negative | | 11 |
| AFP(ng/mL) | ≤20 | | 7 |
|  | >20 | | 69 |
| TNM | I-II | | 53 |
|  | III-IV | | 23 |
| Tumor size(cm) | ≤5 | | 30 |
|  | >5 | | 46 |
| Tumor number | Single | | 56 |
|  | Multiple | | 20 |
| Encapsulation | Complete | | 56 |
|  | None | | 20 |
| Pathologic satellite | Yes | | 31 |
|  | No | | 45 |
| Portal vein tumor thrombus | Yes | | 25 |
|  | No | | 51 |
| BCLC stage | A | | 31 |
|  | B or C | | 45 |

HBsAg, hepatitis B virus surface antigen; AFP, α-fetoprotein; TNM, Tumor-Nodes-Metastasis; BCLC, Barcelona Clinic Liver Cancer Staging.

**Supplementary Table 3. Clinicopathologic Features of 81 HCC Specimens (cohort 2).**

| **Characteristics** | |  | **Number of Patients** |
| --- | --- | --- | --- |
| **Age(year)** | ≤50 | | 37 |
|  | >50 | | 44 |
| **Gender** | Male | | 68 |
|  | Female | | 13 |
| **HBsAg** | Positive | | 67 |
|  | Negative | | 14 |
| **AFP(ng/mL)** | ≤400 | | 50 |
|  | >400 | | 31 |
| **TNM** | I-II | | 68 |
|  | III-IV | | 13 |
| **Tumor size(cm)** | ≤5 | | 38 |
|  | >5 | | 43 |
| **Tumor number** | Single | | 69 |
|  | Multiple | | 12 |
| **Encapsulation** | Complete | | 35 |
|  | None | | 46 |
| **Pathologic satellite** | Yes | | 36 |
|  | No | | 45 |
| **Portal vein tumor thrombus** | Yes | | 8 |
|  | No | | 73 |
| **BCLC stage** | A | | 62 |
|  | B or C | | 19 |

HBsAg, hepatitis B virus surface antigen; AFP, α-fetoprotein; TNM, Tumor-Nodes-Metastasis; BCLC, Barcelona Clinic Liver Cancer Staging.

**Supplementary Table 4. Clinicopathologic Features of 80 HCC Specimens (cohort 3).**

| **Characteristics** | |  | **Number of Patients** |
| --- | --- | --- | --- |
| **Age(year)** | ≤50 | | 47 |
|  | >50 | | 33 |
| **Gender** | Male | | 71 |
|  | Female | | 9 |
| **HBsAg** | Positive | | 75 |
|  | Negative | | 5 |
| **AFP(ng/mL)** | ≤400 | | 52 |
|  | >400 | | 28 |
| **TNM** | I-II | | 28 |
|  | III-IV | | 52 |
| **Tumor size(cm)** | ≤5 | | 17 |
|  | >5 | | 63 |
| **Tumor number** | Single | | 75 |
|  | Multiple | | 5 |
| **Encapsulation** | Complete | | 35 |
|  | None | | 45 |
| **Pathologic satellite** | Yes | | 41 |
|  | No | | 39 |
| **Portal vein tumor thrombus** | Yes | | 41 |
|  | No | | 39 |
| **BCLC stage** | A | | 28 |
|  | B or C | | 52 |

HBsAg, hepatitis B virus surface antigen; AFP, α-fetoprotein; TNM, Tumor-Nodes-Metastasis; BCLC, Barcelona Clinic Liver Cancer Staging.

**Supplementary Table 5. Primers used for qRT-PCR**.

| **Gene** | **Forward primer (5’-3’)** | **Reverse primer (5’-3’)** |
| --- | --- | --- |
| NEAT1 | GGGTGGTCTGAGGAGTGATG | CCTGGAAAATAAAGCGTTGGT |
| EpCAM | TCGCGTTCGGGCTTCTGCTT | GGGCCCCTTCAGGTTTTGCT |
| CD24 | TGAAGAACATGTGAGAGGTTTGAC | GAAAACTGAATCTCCATTCCACAA |
| CYR61 | CAGGACTGTGAAGATGCGGT | AGCCTGTAGAAGGGAAACGC |
| CTGF | CACCCGGGTTACCAATGACA | TCCGGGACAGTTGTAATGGC |
| β-actin | CGTGGACATCCGCAAAG | AAGGTGGACAGCGAGGC |
| 18s | CGGCTACCACATCCAAGGAA | GCTGGAATTACCGCGGCT |

**Supplementary Table 6. Antibody used in this study.**

| **Antigens** | **Manufacturer** | **Application** |
| --- | --- | --- |
| EpCAM-APC | Mitenyi Biotec | 1:50 for FCM |
| AKAP8 | Cell Signaling Technology | 1:1000 for WB  1:100 for IF  5μg for RIP |
| GAPDH | Santa Cruz Biotechnology | 1:5000 for WB |
| GFP | ABclonal Technology | 1:1000 for WB  5μg for RIP |
| Lamin B1 | Proteintech | 1:1000 for WB |
| PKA R2 | Abcam | 1:1000 for WB |
| PKA Cα | Proteintech | 1:1000 for WB |
| p-LATS1/2(Thr1079) | Cell Signaling Technology | 1:1000 for WB |
| LATS1/2 | Cell Signaling Technology | 1:1000 for WB |
| p-YAP(S127) | Abcam | 1:1000 for WB |
| YAP | Abcam | 1:1000 for WB  1:100 for IF  1:100 for IHC |

FCM, Flow cytometry; WB, Western Blot; IF, immnuoflurorescence; RIP, RNA-binding protein immunoprecipitation; IHC, immnuohistochemistry.

**Supplementary Table 7. Primers used for** ChIP-qPCR.

|  | **primer (5’-3’)** |
| --- | --- |
| SB1 forward | AGCGAATGGATCCCACTCTT |
| SB1 reverse | TCACCAGGCATCTGAGAGGT |
| SB2 forward | AAGGTCTGGCTGACTCCACT |
| SB2 reverse | CTGTATCTCCAGGGGTGGGT |
| Neg forward | CACGGTCCAAAGTCCTCTCC |
| Neg reverse | TCTGACATAGCCGAGGGACA |

**Supplementary Table 8. Primers used for** in vitro transcription.

| **NEAT1** | **primer (5’-3’)** |
| --- | --- |
| Sense forward | TAATACGACTCACTATAGGGGGAGTTAGCGACAGGGAGGG |
| Sense reverse | ATAGTTTAGCGGCCGCTTCTAATGAGTTTAGAAC |
| Anti-sense forward | TAATACGACTCACTATAGGGTTCTAATGAGTTTAGAAC |
| Anti-sense reverse | GGGATATCGGAGTTAGCGACAGGGAGGG |

**Supplementary Table 9.** Sequences of siRNA used in this study.

| **Target** | **Sequence (5’-3’)** |
| --- | --- |
| siNEAT1-1 | CTGGTATGTTGCTCTGTATGGTAAG |
| siNEAT1-2 | GTGAGAAGTTGCTTAGAAACTTTCC |
| siYAP-1 | GGTGATACTATCAACCAAATT |
| siYAP-2 | CTGCCACCAAGCTAGATAATT |
| siAKAP8-1 | GCAGAGTCTAAAGACGCTGTT |
| siAKAP8-2 | GCCTGTTCTGTATGCAAGTTT |
| siPKA Cα-1 | GAAATCCGGGTCTCCATCAAT |
| siPKA Cα-2 | GATCGAACACACCCTGAATGA |
